# Using DNA methylation to detect brain metastases before they spread

**DOI:** 10.1097/MS9.0000000000004478

**Published:** 2025-12-04

**Authors:** Mir Raza Ali, Ritu Panjwani, Iman Osman Abufatima

**Affiliations:** a Department of Medicine, Jinnah Postgraduate Medical Centre, Karachi, Pakistan; b Department of Medicine, University of Medical Sciences and Technology, Khartoum, Sudan

*Dear Editor,*

Detecting brain metastases early is still one of the most difficult problems in oncology. These lesions are frequently not detected by conventional imaging until symptoms have manifested, and there are few available treatments. New developments in DNA methylation profiling are providing a promising non-invasive method for detecting metastases considerably earlier^[1,2]^.

Cell-free DNA, or liquid biopsy samples, can now be used to identify tumor methylomes in the circulation, which are distinct DNA methylation patterns from cancer cells^[2,3]^. Even before the cancer spreads noticeably on scans, these methylation patterns can assist in identifying the tissue of origin, as they function as fingerprints of particular tumor types^[1,4]^.

Studies have yielded favorable outcomes. According to a Nature Medicine research study, methylation-based liquid biopsy may accurately discriminate between multiple types of brain cancers using only plasma samples^[1]^. Similarly, Cancer Cell researchers discovered that circulating tumor DNA methylation patterns can detect brain metastases in high-risk cancer patients earlier than traditional imaging approaches^[2,4]^. This method may allow oncologists to start treatment before neurological symptoms appear, thereby increasing survival and quality of life^[3]^.

DNA methylation testing may be used to track therapy response and recurrence, in addition to early detection. Methylation alterations can indicate the advancement of the disease earlier than traditional biomarkers, since they take place early in the development of cancer^[3,5]^. By incorporating these molecular tools into clinical procedures, invasive biopsies may be less necessary, and tailored care may be improved.

There are still obstacles in the way of this technology’s potential. Before general clinical usage, it is crucial to standardize testing procedures, make them affordable, and validate them in a variety of groups^[4,5]^.

With further study, DNA methylation-based detection may soon become a useful component of cancer surveillance plans, representing a significant advancement in precision oncology.

This manuscript complies with TITAN Guidelines, 2025, declaring no use of AI^[6]^.

## Data Availability

Data sharing is not applicable to this article.

## Research registration unique identifying number (UIN)

Our study does not involve any human participants or human data; therefore, registration in a study registry is not applicable.
